# A novel tetra-primer ARMS-PCR approach for the molecular karyotyping of chromosomal inversion 2Ru in the main malaria vectors *Anopheles gambiae* and *Anopheles coluzzii*

**DOI:** 10.1186/s13071-023-06014-6

**Published:** 2023-10-27

**Authors:** Verena Pichler, Antoine Sanou, R. Rebecca Love, Beniamino Caputo, Marco Pombi, Kobie Hyacinth Toe, Moussa W. Guelbeogo, N’Fale Sagnon, Heather M. Ferguson, Hilary Ranson, Alessandra della Torre, Nora J. Besansky

**Affiliations:** 1 Dipartimento di Sanità Pubblica e Malattie Infettive, Istituto Pasteur-Fondazione Cenci-Bolognetti, Università “La Sapienza”, 00185 Rome, Italy; 2 Centre National de Recherche et de Formation Sur le Paludisme, Ouagadougou, Burkina Faso; 3 Institute of Biodiversity, Animal Health & Comparative Medicine, Glasgow University, Glasgow, G128QQ UK; 4 Department of Biological Sciences, University of Notre Dame, Notre Dame, IN 46556 USA; 5 Eck Institute for Global Health, University of Notre Dame, Notre Dame, IN 46556 USA; 6 Department of Vector Biology, Liverpool School of Tropical Medicine, Liverpool, UK; 7 Present Address: Entomology Branch, Division of Parasitic Diseases and Malaria, U.S. Centers for Disease Control and Prevention (CDC), Atlanta, GA 30333 USA

**Keywords:** *Anopheles gambiae* complex, Chromosomal inversion, Inversion genotyping, Malaria vector, Molecular karyotyping, Tetra-primer ARMS-PCR

## Abstract

**Background:**

Chromosomal inversion polymorphisms have been associated with adaptive behavioral, physiological, morphological and life history traits in the two main Afrotropical malaria vectors, *Anopheles coluzzii* and *Anopheles gambiae*. The understanding of the adaptive value of chromosomal inversion systems is constrained by the feasibility of cytological karyotyping. In recent years in silico and molecular approaches have been developed for the genotyping of most widespread inversions (2La, 2Rb and 2Rc). The 2Ru inversion, spanning roughly 8% of chromosome 2R, is commonly polymorphic in West African populations of *An. coluzzii* and *An. gambiae* and shows clear increases in frequency with increasing rainfall seasonally and geographically. The aim of this work was to overcome the constraints of currently available cytological and high-throughput molecular assays by developing a simple PCR assay for genotyping the 2Ru inversion in individual specimens of both mosquito species.

**Methods:**

We designed tetra-primer amplification refractory mutation system (ARMS)-PCR assays based on five tag single-nucleotide polymorphisms (SNPs) previously shown to be strongly correlated with 2Ru inversion orientation. The most promising assay was validated against laboratory and field samples of *An. coluzzii* and *An. gambiae* karyotyped either cytogenetically or molecularly using a genotyping-in-thousands by sequencing (GT-seq) high-throughput approach that employs targeted sequencing of multiplexed PCR amplicons.

**Results:**

A successful assay was designed based on the tag SNP at position 2R, 31710303, which is highly predictive of the 2Ru genotype. The assay, which requires only one PCR, and no additional post-PCR processing other than electrophoresis, produced a clear banding pattern for 98.5% of the 454 specimens tested, which is a 96.7% agreement with established karyotyping methods. Sequences were obtained for nine of the *An. coluzzii* specimens manifesting 2Ru genotype discrepancies with GT-seq. Possible sources of these discordances are discussed.

**Conclusions:**

The tetra-primer ARMS-PCR assay represents an accurate, streamlined and cost-effective method for the molecular karyotyping of the 2Ru inversion in *An. coluzzii* and *An. gambiae.* Together with approaches already available for the other common polymorphic inversions, 2La, 2Rb and 2Rc, this assay will allow investigations of the adaptive value of the complex set of inversion systems observed in the two major malaria vectors in the Afrotropical region.

**Graphical Abstract:**

**Figure:**
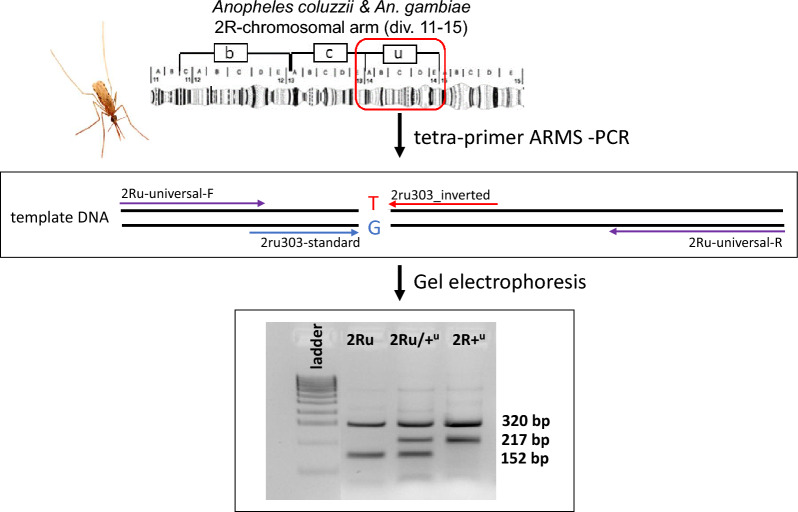

**Supplementary Information:**

The online version contains supplementary material available at 10.1186/s13071-023-06014-6.

## Background

The tropical African *Anopheles gambiae* complex comprises at least nine species that are morphologically indistinguishable but which vary widely in terms of their medical importance owing to ecological and behavioral differences [1, 2]. Chromosomal inversions, described as structural rearrangements involving breakage and reversal of a chromosome segment, are prevalent but nonrandomly distributed in this complex, being disproportionately abundant in the three species that are the primary vectors of human malaria [2, 3]. Historically, the few inversions found as fixed differences between species have assumed a practical importance for species identification [2], enabling ecological and epidemiological studies that have defined malaria vectorial roles. Yet, understanding of the importance of the  > 120 polymorphic inversions observed in natural populations remains largely incomplete [3, 4].

Chromosomal inversions are recombination modifiers [5]. The best understood mechanism involves strongly reduced recombination between opposite orientations in inversion heterozygotes, caused by gross gametic aneuploidies in meiotic crossover products [5]. Reduced recombination preserves favorable combinations of locally beneficial alleles as haplotype blocks, which are protected against genomic homogenization with maladapted genetic backgrounds [6]. The local adaptation hypothesis proposes that such recombination suppression inside chromosomal inversions plays a key role in local adaption, ecotype formation and speciation in the face of gene flow [6, 7]. Empirical evidence consistent with this hypothesis is mounting from diverse species of plants and animals, including the *An. gambiae* complex [8–14].

The sister taxa *An. gambiae* and *Anopheles coluzzii* are both characterized by extraordinary ecological flexibility [15, 16]. They have successfully colonized diverse natural habitats across sub-Saharan Africa as well as areas characterized by anthropogenic environmental modifications associated with agricultural development and urbanization [17–19]. The dominance of the two species across geographically and seasonally heterogeneous habitats, a situation related to their extensive inversion polymorphism [3, 18, 19], is a major factor in their status as the most efficient malaria vectors worldwide. Recurrent seasonal fluctuations and stable latitudinal or altitudinal clines in the frequencies of most of the common chromosome 2 inversions (2La, 2Rb, 2Rc, 2Rd, 2Ru) in relation to rainfall, documented in multiple parts of Africa, implicate spatially varying selection in the maintenance of inversion polymorphism [2, 12, 18, 19]. The two most geographically widespread and best-studied inversions, 2La and 2Rb, have been associated with a number of adaptive behavioral, physiological, morphological and life history traits conferring aridity tolerance [20–25]. However, further understanding of adaptive inversion polymorphism systems [26–28] will require additional genetic, ecological and modeling studies of natural populations.

A major barrier to further progress in the understanding of the adaptive value of inversion systems in the *An. gambiae* complex has been logistical. Until recently, chromosomal inversions could be studied only by expert cytogenetic analysis of polytene chromosomes. Both the paucity of specialized cytogenetic expertise and the labor-intensive nature of preparing and scoring chromosomes imposed severe limitations, much exacerbated by the requirement of live or appropriately preserved adult female mosquitoes at the correct gonotrophic stage to obtain favorable ovarian polytene chromosomes.

Significant advances in genomic technology and analysis have alleviated these limitations, even for organisms that lack polytene chromosomes or metaphase chromosomes favorable for cytogenetic analysis—assuming access to population-level individual whole-genome sequencing (see, for example [8, 10]). For *An. gambiae* and *An. coluzzii*, an in silico approach was recently developed for the genotyping of multiple inversions in individual fully sequenced mosquitoes, based on tag single-nucleotide polymorphisms (SNPs) highly predictive of inversion orientation [29]. In addition, based on these same tag SNPs, high-throughput molecular assays have been developed that are capable of genotyping multiple inversions simultaneously in hundreds or thousands of individual mosquitoes, either without sequencing (using a genotyping array) or through targeted sequencing of multiplexed PCR amplicons [30]. However, in the absence of whole-genome sequence data, or where a high-throughput molecular approach is inappropriate due to budget constraints or scientific scope, it is highly desirable to have robust and accurate PCR-based assays for genotyping individual inversions in individual mosquitoes. Such an assay has long existed for genotyping inversion 2La in the *An. gambiae* complex [31]. This robust assay was developed based on precise molecular characterization of the inversion breakpoints, a rare achievement due to the frequent association of repetitive DNA with inversion breakpoints. This assay also has the advantage of requiring only three breakpoint-crossing primers in a single PCR assay, without the need of further downstream steps other than electrophoresis [31]. Individual PCR-based inversion genotyping assays that are both robust and accurate have been recently developed for 2Rb [32] and 2Rc [33], although they have some operational drawbacks. In both cases, achieving the highest level of accuracy requires performing two separate reactions, and genotyping requires the additional step of subjecting the PCR amplicon to restriction digestion prior to electrophoresis. Moreover, the 2Rc genotyping assay works well only for *An. coluzzii*.

**Fig. 1:**
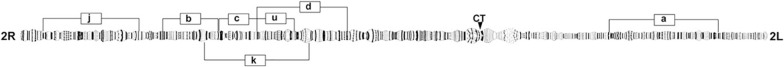
Diagrammatic representation of the 2Ru chromosomal inversion and other common polymorphic inversions, shown as lowercase letters in boxes on chromosomal arm 2R in *Anopheles coluzzii* and *Anopheles gambiae*. Polytene chromosome map is modified from Fig. 1 and poster in [3]. CT, Centromere

The 2Ru inversion spans approximately 4 Mb—roughly 8%—of chromosome 2R euchromatin [3, 29] (Fig. 1). It is commonly polymorphic in West African populations of *An. coluzzii* and *An. gambiae* [34]*,* and shows clear increases in frequency with increasing rainfall, seasonally and geographically, consistent with a role in climatic adaptation [2, 18, 19]. Before now, no individual molecular genotyping assay was available for 2Ru. Here, we develop a novel tetra-primer amplification refractory mutation system-PCR (ARMS-PCR) assay for the genotyping of the 2Ru inversion of both *An. gambiae* and *An. coluzzii*. This genotyping assay requires only one PCR reaction, and no additional post-PCR processing other than electrophoresis. This rapid, accurate and cost-effective 2Ru assay enables investigations into the role of 2Ru in local adaptation in the *An. gambiae* complex.

## Methods

### Tetra-primer ARMS-PCR assay design

**Fig. 2:**
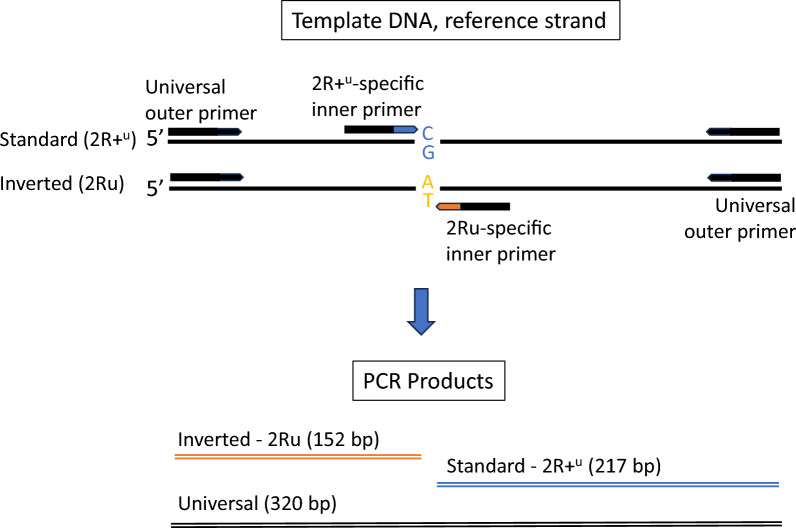
Scheme and expected results of inversion 2Ru-specific tetra-primer ARMS-PCR designed for locus 2R:31710303. ARMS–PCR, Amplification refractory mutation system-PCR

The tetra-primer ARMS-PCR is an approach to SNP genotyping that involves a single PCR followed by agarose gel electrophoresis [35]. It entails the use of four primers: two outer ‘universal’ (non-allele-specific) primers that amplify the region containing the SNP, and two allele-specific inner primers targeting alternative alleles at a diagnostic SNP (Table 1). Non-allele-specific template amplification by the outer universal primers creates a positive control PCR amplicon. Allele specificity of the inner primers is achieved not only by designing the 3′—terminus to bind the alternative alleles of the tag SNP, but also by incorporating a deliberate mismatch at the third 3′—terminal base of the primer. Placement of the universal primers at sufficiently different distances from the target SNP allows the allele-specific amplicons to be distinguished electrophoretically (Fig. 2). Therefore, only a single PCR is necessary to discriminate the two alleles at the target locus instead of two separate reactions (one for each allele) as required by conventional allele-specific (AS) PCR assays.

**Table 1:** Primers for the tetra-primer amplification refractory mutation system-PCR targeting the 2Ru tag single-nucleotide polymorphism 2R:31710303 in *Anopheles coluzzii* and *Anopheles gambiae*

Primer name	SNP target	2Ru genotype^a^	Sequence 5′—3′^b^, ^c^
2Ru-universal-F	Non-allele-specific	Non-specific	GATGATACGGATTTGCTGGCAAG
2Ru-universal-R	Non-allele-specific	Non-specific	GGAATGTGTGAAAATGTGCCTCCACTG
2Ru-inverted	Allele T, 2R:31,710,303	2Ru	AGANGAAGAAAATGCTCTCGCNT*T*GA
2Ru-standard	Allele G, 2R:31,710,303	2R+^u^	CAAGCAACTGGCGTCGAAGTNAA*G*TG

*SNP* Single-nucleotide polymorphism

^a^2Ru/+^u^, Heterozygote

^b^Nucleotides in italics and underlined within the internal primers identify intentionally inserted mismatches

^c^An ‘N’ denotes any nucleotide

Regarding the design of the 2Ru assay, we began with a set of 177 tag SNPs previously reported to be highly predictive of 2Ru inversion orientation (see Table 1 in [29]), based on genomic sequence variation in *An. gambiae* and *An. coluzzii* natural populations represented in the Ag1000G catalog (http://www.malariagen.net/mosquito/ag1000g; [36]). Mosquito samples in the Ag1000G catalog were used to assess concordance between SNPs and the 2Ru genotype represented in multiple African countries (see Additional file 1: Table S2 of [29]). Of the 177 candidate tag SNPs predictive of 2Ru, we focused on five among those SNPs showing the highest degree of concordance with inversion genotype (> 97.8%; 2R:31710303, 2R:34739085, 2R:34739416, 2R:34739767, 2R:35498331).

Primer design and assay optimization for the candidate 2Ru tag SNPs followed published guidelines [35]. Primer placement and design were informed with reference to the *An. gambiae* PEST genome assembly AgamP4, accessed through VectorBase (https://vectorbase.org/vectorbase/app; [37]). If primer binding sites spanned SNPs segregating at frequencies > 5% in Ag1000G, primers were synthesized with an “*N*” at those positions. Primers were designed using the web service PRIMER1 (http://primer1.soton.ac.uk/primer1.html; [38]). The specificity of primers was checked using BLAST (https://blast.ncbi.nlm.nih.gov/Blast.cgi).

**Fig. 3:**
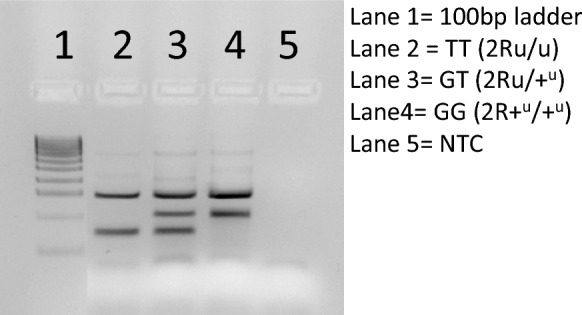
Representative electrophoretic profile of the 2Ru-specific tetra-primer ARMS-PCR assay for locus 2R:31710303 showing an inverted homozyogte (2Ru/u; lane 2), a heterozygote (2Ru/+^u^; lane 3) and a standard (un-inverted) homozygote (2R+^u^/+^u^; lane 4). Lane 1 shows the molecular weight marker (HyperLadder 100 bp; Bioline, Memphis, TN, USA): 100–1000 bp in increments of 100 bp. NTC = No-template-control; ARMS-PCR, Amplification refractory mutation system-PCR

Each PCR assay was carried out in a 25-μl reaction volume containing 1 U of Taq polymerase (Bioline, Memphis, TN, USA), 10× PCR Buffer (Bioline), 0.1 mM of each dNTP, 2.5 mM MgCl_2_, 0.2 µM of each outer primer, 1 µM of each inner allele-specific primer and 1 μl of template genomic DNA, on a Bio-Rad C1000 Touch thermal cycler (Bio-Rad Laboratories, Hercules, CA, USA). The PCR cycling conditions consisted of an initial incubation at 95 °C for 3 min; 35 cycles of 95 °C for 30 s, 62 °C for 30 s and 72 °C for 40 s; followed by 72 °C for 5 min and a 10 °C hold. The PCR amplification products were then subjected to gel electrophoresis (30–40 min at 90 V in 1× TBE buffer) in 3% agarose gels and stained with Midori Green Advance (Nippon Genetics, Tokyo, Japan) (Fig. 3).

### Tetra-primer ARMS-PCR assay validation

The tetra-primer ARMS-PCR assay for 2Ru was validated against *An. coluzzii* and *An. gambiae* samples karyotyped by one of two proven methods.

Previous cytogenetic karyotyping was performed as described in [32] on 28 *An. coluzzii*, 40 *An. gambiae* and one *An. coluzzii—An. gambiae* hybrid sampled from natural populations in Benin (*N* = 19), Mali (*N* = 8), Senegal (*N* = 28) and the Democratic Republic of Congo (*N* = 14). An additional 18 *An. coluzzii* specimens of the Banfora-M colony from Burkina Faso, obtained from the Liverpool School of Tropical Medicine and Hygiene, were also cytogenetically karyotyped.

In addition, we obtained 367 *An. coluzzii* adult females sampled by human landing catch in August of 2017 in the village of Sitiena in south-western Burkina Faso (10.6º N − 4.8º W). DNA was extracted from individual mosquitoes using a CTAB method [39] and identified at species level using the SINE200 PCR assay [40]. A high-throughput molecular approach that employs targeted sequencing of multiplexed PCR amplicons (genotyping-in-thousands by sequencing [GT-seq]; [41]) was used for inversion genotyping, as previously described [30]. This method, which predicts the inversion genotype based on averaging across multilocus tag SNPs that are individually highly predictive, has been shown to be comparable or superior to traditional cytogenetic karyotyping [30]. In the case of disagreement between the 2Ru genotype indicated by the tetra-primer ARMS-PCR assay versus the GT-seq genotype, a PCR assay was performed using only the outer (non-allele-specific) primers (Table 1). The resulting amplicon was purified using the SureClean Kit (Bioline) and sequenced at BMR Genomics s.r.l. (Padua, Italy; Additional file 1: Text S1).

## Results and discussion

A tetra-primer ARMS-PCR assay was successfully designed based on the tag SNP at position 2R:31710303 that is highly predictive of the 2Ru genotype (Fig. 2) using the primer sequences provided in Table 1. In contrast, the design of a tetra-primer ARMS-PCR approach for the remaining four SNPs was unsuccessful.

The novel tetra-primer ARMS-PCR assay was validated on a total of 454 specimens karyotyped by proven methods, either by traditional cytogenetic analysis or by a high-throughput multilocus GT-seq approach [30]. Overall, the PCR assay produced a clear banding pattern for 98.5% of the specimens, with a concordance of 96.7% between the new assay and established methods.

Of the specimens with cytogenetically determined 2Ru karyotypes, 83 (of 87) were successfully genotyped using the tetra-primer ARMS-PCR assay, and there was 100% agreement on the obtained inversion genotype between methods (Table 2). Of the 367 Burkina Faso specimens with GT-seq-derived 2Ru inversion genotypes, 365 were successfully genotyped with the new PCR assay. Concordance between the results from GT-seq and those from the tetra-primer ARMS-PCR was 95.9% (Table 2; Additional file 1: Table S1). For the 15 *An. coluzzii* specimens manifesting discrepancies in the 2Ru genotype between methodological approaches, the tetra-primer ARMS-PCR was repeated, with unchanged results. Most (12/15) of these disagreements involved heterozygous calls by GT-seq versus homozygous calls by the tetra-ARMS-PCR assay (2R+^u^/+^u^ or 2Ru/u), without a clear directional bias.

**Table 2:** Performance of the tetra-primer amplification refractory mutation system-PCR-PCR 2Ru genotyping assay versus proven 2Ru genotyping methods (genotyping-in-thousands by sequencing and cytogenetic karyotyping

Karyotyping method	2Ru inversion genotype^a^	Tetra-primer ARMS-PCR^b^			Concordance %
		2R+^u^/+^u^	2Ru/+^u^	2Ru/u	
GT-seq	2R+^u^/+^u^	238	–	–	95.9
	2Ru/+^u^	*7*	92	*5*	
	2Ru/u	-	*3*	20	
Cytogenetic karyotyping	2R+^u^/+^u^	76	–	–	100
	2Ru/+^u^	–	5	–	
	2Ru/u	–	–	2	

*ARMS* Amplification refractory mutation system,* GT-seq *genotyping-in-thousands by sequencing

^a^2R+^u^/+^u^, Standard (un-inverted) homozygote; 2Ru/+^u^, heterozygote; 2Ru/u, inverted homozyogte

^b^Numbers in italics show discordance in the results of the two methods

To investigate the basis for the discrepant genotypes, template DNA from each of the 15 mosquito specimens included in the study was subjected to PCR using only the universal (non-allele-specific) outer primers, followed by sequencing of the resulting 320-bp amplicon. Readable sequences were obtained for nine specimens (Additional file 1: Table S2). In one of these nine specimens, sequencing resolved the conflict by revealing that the 320-bp universal amplicon of the tetra-primer ARMS-PCR assay was heterozygous for the 2Ru tag SNP indicative of an inversion heterozygote (2Ru/+^u^), in agreement with the GT-seq results, and not of an inverted homozygote (2Ru/u), as the electrophoretic results from the assay had suggested. In this case, it would appear that the internal allele-specific primer targeting the standard (uninverted) orientation of the 2Ru inversion did not bind efficiently, despite the fact that we detected no polymorphisms or substitutions in the allele-specific primer binding sites.

For the remaining eight specimens with readable sequences, sequencing of the 320-bp universal amplicon did not resolve the conflict with GT-seq; instead, the data confirmed the electrophoretic tetra-primer ARMS-PCR genotype. As previously reported [29], any individual tag SNP is almost never perfectly associated with the inversion orientation, owing primarily to rare double crossovers or gene conversion between inversion orientations in heterozygotes. However, in considering the most likely basis for disagreements between genotyping methods, it is important to emphasize that the GT-seq approach is highly robust to deviations from perfect concordance between allelic state and inversion orientation at any individual tag SNP because it genotypes inversion orientation based on the combined results from multiple tag SNPs (17 in the case of 2Ru), rather than on the single SNP detected by the tetra-primer ARMS-PCR assay [30]. Accordingly, if the same mosquito template DNA yields discordant 2Ru genotypes between the two methods, the most likely culprit for the discordance is the single-SNP PCR assay. Under the reasonable assumption that GT-seq can be considered a gold standard for 2Ru genotyping, there are two non-exclusive and plausible explanations for the few ‘failures’ of the tetra-primer ARMS-PCR assay. First and most obvious, the set of 17 tag SNPs for 2Ru assessed by GT-seq does not include the additional tag SNP (SNP 2R:31710303) targeted by the tetra-primer ARMS-PCR assay. The latter SNP had a strong (98%) yet imperfect association with 2Ru inversion orientation in the Ag1000G variation database [29]. The imperfect association between this single SNP and inversion orientation may explain some or even most discrepancies.

Another credible explanation that could contribute to the few apparent failures of the tetra-primer ARMS-PCR assay is a known limitation of PCR-based molecular diagnostic approaches more generally, termed allelic dropout [42]. The total loss or massive underrepresentation of one allele during PCR amplification of DNA in a PCR-based assay can be caused by common or rare point mutations in primer binding sites, resulting in an overrepresentation of homozygotes. Natural populations of both *An. gambiae* and *An. coluzzii* typically carry exceptionally high levels of genetic diversity [36]. The fact that we observed a large majority of discordant genotypes in which GT-seq predicted a 2Ru heterozygote while the tetra-primer ARMS-PCR assay called a homozygote may implicate allelic dropout.

Although the new tetra-primer ARMS-PCR assay for 2Ru is not infallible, its performance compared to proven methods of inversion genotyping is very strong: nearly 97% concordance between methods across 454 genotyped specimens. For perspective, it has been estimated that the rate of erroneous interpretations of the polytene complement when cytogenetic karyotyping is performed by a highly experienced cytogeneticist is 4% (V. Petrarca, personal communication).

Compared to other studies validating molecular karyotyping PCR-approaches for more widespread inversions, such as 2La [31] and 2Rb [32], the weaknesses of the present study include underrepresentation of *An. gambiae* in our sampling (*N* = 40), the low number of 2Ru/u homozygotes available for validation (*N* = 23) and the relatively limited geographic sampling of *An. gambiae* and *An. coluzzii* populations. However, it is relevant to note that the taxonomic distribution of the 2Ru inversion is not evenly balanced between taxa. Its prevalence is higher in *An. coluzzii* than in *An. gambiae* [15, 16, 34], where it is generally present at low frequencies except in Mali [19]. Indeed, of the *An. gambiae* represented in Ag1000G that were used to ascertain tag SNPs for the 2Ru inversion, nearly all were sourced from Mali [29]. Furthermore, the geographic distribution of 2Ru is confined to West Africa in both taxa [34]. Although our geographic sampling was not comprehensive, the association of tag SNP 2R:31710303 used in the new assay with 2Ru inversion orientation was detected and validated in more than 1100 specimens of both *An. coluzzii* and *An. gambiae* from 11 African countries [29], lending confidence that the tetra-ARMS-PCR assay for 2Ru will provide a reasonably accurate 2Ru genotype for both species across the range of the inversion.

In conclusion, we have shown that the tetra-primer ARMS-PCR assay represents an accurate, streamlined and cost-effective method for the molecular karyotyping of the 2Ru inversion in *An. coluzzii* and *An. gambiae.* Together with other approaches already available for the other common polymorphic inversions 2La, 2Rb and 2Rc, this assay will allow investigations of the adaptive value of the complex set of inversion systems observed in the two major malaria vectors in the Afrotropical region. Future efforts should be devoted to extending the tetra-primer ARMS-PCR approach to these other inversions, and multiplexing the assays, which would further simplify and encourage ecological and evolutionary studies of inversion polymorphism in this medically important group.

### Supplementary Information

**Additional file 1: Table S1.** Comparison between results of 2Ru inversion genotyping either by cytogenetics (in rows) or by tetra-primer ARMS-PCR (in columns) in field-collected *An*. *coluzzii*, *An*. *gambiae* and hybrid specimens from West Africa and in laboratory samples. **Table S2.** Sequencing results obtained for field* Anopheles coluzzii* specimens from Burkina Faso genotyped discordantly by the multilocus GT-seq approach and the 2Ru tetra-primer ARMS-PCR. Concordant results in green. **Text S1.** Alignment in fasta format of sequences obtained for field-collected* Anopheles coluzzii* specimens from Burkina Faso with discordant genotypes by the multilocus GT-seq approach and the 2Ru tetra-primer ARMS-PCR.

## Data Availability

All data analyzed in the manuscript are available within the manuscript and its Additional file material.
