# Supplementary material for: A novel tetra-primer ARMS-PCR approach for the molecular karyotyping of chromosomal inversion 2Ru in the main malaria vectors Anopheles gambiae and Anopheles coluzzii
Source: Parasit Vectors. 2023 Oct 27;16:388. doi: 10.1186/s13071-023-06014-6 (PMC10605393; doi:10.1186/s13071-023-06014-6)
Supplement: Supplementary file 1 — Additional file 1: Table S1. Comparison between results of 2Ru inversion genotyping either by cytogenetics (in rows) or by tetra-primer ARMS-PCR (in columns) in field-collected An. coluzzii, An. gambiae and hybrid specimens from West Africa and in laboratory samples. Table S2. Sequencing results obtained for field Anopheles coluzzii specimens from Burkina Faso genotyped discordantly by the multilocus GT-seq approach and the 2Ru tetra-primer ARMS-PCR. Concordant results in green. Text S1. Alignment in fasta format of sequences obtained for field-collected Anopheles coluzzii specimens from Burkina Faso with discordant genotypes by the multilocus GT-seq approach and the 2Ru tetra-primer ARMS-PCR. [file 13071_2023_6014_MOESM1_ESM.docx]

## Additional file 1

**Table S1**. **Comparison between results of 2Ru inversion genotyping either by cytogenetics (in rows) or by tetra-primer ARMS-PCR (in columns)** in field collected *An. coluzzii, An. gambiae* and hybrid specimens from West Africa and in laboratory samples.

|  |  |  | **Tetra-ARMS PCR assay** | | |
| --- | --- | --- | --- | --- | --- |
| **Species** | **country** | **Cytogenetic karyotping** | 2R+^u^/+^u^ | 2Ru/+^u^ | 2Ru/u |
| ***An.coluzzii*** | **Benin^1^** | 2R+^u^/+^u^ | 18 | - | - |
|  |  | 2Ru/+^u^ | - | - | - |
|  |  | 2Ru/u | - | - | - |
|  | **Mali^2^** | 2R+^u^/+^u^ | - | - | - |
|  |  | 2Ru/+^u^ | - | 5 | - |
|  |  | 2Ru/u | - | - | 1 |
|  | **Senegal^3^** | 2R+^u^/+^u^ | 2 | - | - |
|  |  | 2Ru/+^u^ | - | - | - |
|  |  | 2Ru/u | - | - | - |
|  | **Banfora lab colony** | 2R+^u^/+^u^ | 18 | - | - |
|  |  | 2Ru/+^u^ | - | - | - |
|  |  | 2Ru/u | - | - | - |
| ***An.gambiae*** | **Mali^2^** | 2R+^u^/+^u^ | - | - | - |
|  |  | 2Ru/+^u^ | - | - | - |
|  |  | 2Ru/u | - | - | 1 |
|  | **Senegal^3^** | 2R+^u^/+^u^ | 23 | - | - |
|  |  | 2Ru/+^u^ | - | - | - |
|  |  | 2Ru/u | - | - | - |
|  | **Democratic Republic of Congo^4^** | 2R+^u^/+^u^ | 14 | - | - |
|  |  | 2Ru/+^u^ | - | - | - |
|  |  | 2Ru/u | - | - | - |
| **Hybrid** | **Senegal^3^** | 2R+^u^/+^u^ | 1 | - | - |
|  |  | 2Ru/+^u^ | - | - | - |
|  |  | 2Ru/u | - | - | - |

**^1^** Akogbeto, M., 1995 Entomological study on malaria transmission in coastal and lagoon areas: the case of a village built on a brackish lake. Ann. Soc. Belg. Med. Trop. 75: 219-227.

**^2^** Coulibaly, M. B., M. Pombi, B. Caputo, D. Nwakanma, M. Jawara et al., 2007 PCR-based karyotyping of *Anopheles gambiae* inversion 2Rj identifies the BAMAKO chromosomal form. Malar. J. 6: 133.

^3^ Petrarca, V., J. Vercruysse, and M. Coluzzi, 1987 Observations on the *Anopheles gambiae* complex in the Senegal River Basin, West Africa. Med. Vet. Entomol. 1: 303-312.

^4^ Petrarca V, personal communication

**Table S2**: **Sequencing results obtained for field Anopheles coluzzii specimens from Burkina Faso genotyped discordantly by the multilocus GT-seq approach and the 2Ru tetra-primer ARMS-PCR.** Concordant results in green.

|  | **2Ru genotype** | | |  |
| --- | --- | --- | --- | --- |
| **ID specimen** | **GT-seq** | **Tetra-ARMS-PCR** | **Universal amplicon sequence** | **Note** |
| 12705-1 | u/+^u^ | +^u^/+^u^ | +^u^/+^u^ | nearby polymorphisms |
| 12706-9 | u/+^u^ | +^u^/+^u^ | +^u^/+^u^ | - |
| 12062-9 | u/+^u^ | +^u^/+^u^ | +^u^/+^u^ | nearby polymorphisms |
| 12063-17 | u/+^u^ | +^u^/+^u^ | +^u^/+^u^ | - |
| 12074-7 | u/+^u^ | +^u^/+^u^ | +^u^/+^u^ | - |
| 12092-1 | u/+^u^ | +^u^/+^u^ | +^u^/+^u^ | - |
| 12717-15 | u/+^u^ | u/u | u/u | nearby polymorphisms |
| 12072-4 | u/+^u^ | u/u | u/+^u^ | - |
| 12206-3 | u/u | u/+^u^ | u/+^u^ | nearby polymorphisms |

**Additional file 1: text S1: Alignment in fasta format of sequences obtained for field Anopheles coluzzii specimens from Burkina Faso** genotyped discordantly by the multilocus GT-seq approach and the 2Ru tetra-primer ARMS-PCR.

>12705-1

GTGGYATGGTTTGGTTGRGTTGCTTTTTCAAATCAAGCAACTGGCGTCGAAGTCAARTGCGACGCGAGASCATTTTCTWCCTCTTACATTCACTCCTTCCAATCCAATCCCAAAGGGAGCAGCCTCGGAGATGGTCGCAATGGTGGTAGTATGTGGATTAAAGATGTGTACYCATGAATGGG

>12706-9

GTGGCAGGGTTTGGTTGAGTTGCTTTTTCAAATCAAGCAACTGGCGTCGAAGTCAAATGCGACGCGAGAGCATTTTMTTCCTCTTACATTCACTCCTTYCAATCCAATCCCAAAGGGAGCAGCCTYGGAGATGGTCGCAATGGTGGTAGTATGTGGATTAAAGATGTGTACYCATGAATGGG

>12717-15

GTGGCAGGGTTTGGTTGAKTTGCTTTTTCAAATCAAGCAACTGGCGTCGAAGTCAAAC

TCAMCGAGAGAGCATTTTCTTCCTCTTACCTTCACTCCTTCCAATCCAATCCCAAAGGGAGCAGCCGCGGAGATGGTCGCAATGGTGGTAGTATGTGGATTAAAGATGTGTACCCATGAATGGG

>12062-9

GTGGCAGGGTTTGGTTGAGTTGCTTTTTCAAATCAAGCAACTGGCGTCGAAGTCAAAYGCRACGCGAGAGCATTTTCTKCCTCTTACATTCACTCCTTCCAATCCAATCCCAAAGGGAGCAGCCTCGGAGATRSTCGCAATGGTGGTAGTATGTGGATTAAAGATGTGTACYCATGAATGGG

>12063-17

GTGGCAGGGTTTGGTTGAGTTGCTTTTTCAAATCAAGCAACTGGCGTCGAAGTCAAATGCGACGCGAGAGCATTTTCTTCCTCTTACATTCACTCCTTCCAATCCAATCCCAAAGGGAGCAGCCTCGGAGATGGTCGCAATGGTGGTAGTATGTGGATTAAAGATGTGTACTCATGAATGGG

>12072-4

GTGGCAGGGTTTGGTTGAGTTGCTTTTTCAAATCAAGCAACTGGCGTCGAAGTCAAACKCAAMGMGAGAGCATTTTCTKCCTCTTACMTTCACTCCTTCCAATCCAATCACACCGGGAGCAGNCCNNNNNNNNNNNNNNNNNNNNNNNNNNNNNNNNNNNNNNNNNNNNNNNNNNNNNNNNN

>12074-7

GTGGCAGGGTTTGGTTGAGTTGCTTTTTCAAATCAAGCAACTGGCGTCGAAGTCAAATGCGACGCGAGAGCATTTTCTKCCTCTTACATTCACTCCTTCCAATCCAATCCCAAAGGGWGCAGCCTCGGAGATGGTCGCAATGGTGGTAGTATGTGGATTAAAGATGTGTACYCATGAATGGG

>12092-1

GTGGYATGGTTTGGTTGRGTTGCTTTTTCAAATCAAGCAACTGGCGTCGAAGTCAAATGCGACGCGAGAGCATTTTCTKCCTCTTACATTCACTCCTTCCAATCCAATCCCAAAGGGAGCAGCCTCGGAGATGGTCGCAATGGTGGTAGTATGTGGATTAAAGATGTGTACYCATGAATGGG

>12206-3

GTGGCAGGGTTTGGTTGAGTTGCTTTTTCAAATCAAGCAACTGGCGTCGAAGTCAAAYKCGACGMGAGAGCATTTTCTTCCTCTTACMTTCACTCCTTCCAATCCAATCCCAAAGGGAGCAGCCKCGGAGATGGTCGCAATGGTGGTAGTATGTGGATTAAAGATGTGTACYCATGAATGGG
